# EMR-linked GWAS study: investigation of variation landscape of loci for body mass index in children

**DOI:** 10.3389/fgene.2013.00268

**Published:** 2013-12-03

**Authors:** Bahram Namjou, Mehdi Keddache, Keith Marsolo, Michael Wagner, Todd Lingren, Beth Cobb, Cassandra Perry, Stephanie Kennebeck, Ingrid A. Holm, Rongling Li, Nancy A. Crimmins, Lisa Martin, Imre Solti, Isaac S. Kohane, John B. Harley

**Affiliations:** ^1^Center for Autoimmune Genomics and Etiology, Cincinnati Children’s Hospital Medical CenterCincinnati, OH, USA; ^2^Cincinnati Children’s Hospital Medical CenterCincinnati, OH, USA; ^3^School of Medicine, University of CincinnatiCincinnati, OH, USA; ^4^Division of Biomedical Informatics, Cincinnati Children’s Hospital Medical CenterCincinnati, OH, USA; ^5^Division of Genetics and Genomics, Boston Children’s HospitalBoston, MA, USA; ^6^Division of Genetics and Genomics, Department of Pediatrics, The Manton Center for Orphan Disease Research, Boston Children’s Hospital, Harvard Medical SchoolBoston, MA, USA; ^7^National Human Genome Research Institute, National Institutes of HealthBethesda, MD, USA; ^8^Center for Biomedical Informatics, Harvard Medical School and Children’s Hospital Informatics ProgramBoston, MA, USA; ^9^Department of Veteran Affairs Medical CenterCincinnati, OH, USA

**Keywords:** BMI, obesity, polymorphism, GWAS

## Abstract

Common variations at the loci harboring the fat mass and obesity gene (FTO), MC4R, and TMEM18 are consistently reported as being associated with obesity and body mass index (BMI) especially in adult population. In order to confirm this effect in pediatric population five European ancestry cohorts from pediatric eMERGE-II network (CCHMC-BCH) were evaluated.

**Method:** Data on 5049 samples of European ancestry were obtained from the Electronic Medical Records (EMRs) of two large academic centers in five different genotyped cohorts. For all available samples, gender, age, height, and weight were collected and BMI was calculated. To account for age and sex differences in BMI, BMI *z*-scores were generated using 2000 Centers of Disease Control and Prevention (CDC) growth charts. A Genome-wide association study (GWAS) was performed with BMI *z*-score. After removing missing data and outliers based on principal components (PC) analyses, 2860 samples were used for the GWAS study. The association between each single nucleotide polymorphism (SNP) and BMI was tested using linear regression adjusting for age, gender, and PC by cohort. The effects of SNPs were modeled assuming additive, recessive, and dominant effects of the minor allele. Meta-analysis was conducted using a weighted *z*-score approach.

**Results:** The mean age of subjects was 9.8 years (range 2–19). The proportion of male subjects was 56%. In these cohorts, 14% of samples had a BMI ≥95 and 28 ≥ 85%. Meta analyses produced a signal at 16q12 genomic region with the best result of *p* = 1.43 × 10^-^^7^ [*p*_(rec)_ = 7.34 × 10^-^^8^) for the SNP rs8050136 at the first intron of FTO gene (*z* = 5.26) and with no heterogeneity between cohorts (*p* = 0.77). Under a recessive model, another published SNP at this locus, rs1421085, generates the best result [*z* = 5.782, *p*_(rec)_ = 8.21 × 10^-^^9^]. Imputation in this region using dense 1000-Genome and Hapmap CEU samples revealed 71 SNPs with *p* < 10^-^^6^, all at the first intron of FTO locus. When hetero-geneity was permitted between cohorts, signals were also obtained in other previously identified loci, including MC4R (rs12964056, *p* = 6.87 × 10^-^^7^, *z* = -4.98), cholecystokinin CCK (rs8192472, *p* = 1.33 × 10^-^^6^, *z* = -4.85), Interleukin 15 (rs2099884, *p* = 1.27 × 10^-^^5^, *z* = 4.34), low density lipoprotein receptor-related protein 1B [LRP1B (rs7583748, *p* = 0.00013, *z* = -3.81)] and near transmembrane protein 18 (TMEM18) (rs7561317, *p* = 0.001, *z* = -3.17). We also detected a novel locus at chromosome 3 at COL6A5 [best SNP = rs1542829, minor allele frequency (MAF) of 5% *p* = 4.35 × 10^-^^9^, *z* = 5.89].

**Conclusion:** An EMR linked cohort study demonstrates that the BMI-*Z* measurements can be successfully extracted and linked to genomic data with meaningful confirmatory results. We verified the high prevalence of childhood rate of overweight and obesity in our cohort (28%). In addition, our data indicate that genetic variants in the first intron of FTO, a known adult genetic risk factor for BMI, are also robustly associated with BMI in pediatric population.

## INTRODUCTION

The electronic MEdical Records and GEnomics (eMERGE) Network, founded in 2007, is a consortium of multiple adult and pediatric institutions developed to explore the utility of DNA bio repositories linked to electronic medical records (EMR) in advancing genomic medicine (McCarty et al., 2011). For each site, the primary site-specific phenotypes have undergone genome-wide association studies (GWAS) with data and results shared through the network. In the pediatric population, however, genetic studies are challenging due to different developmental phase and growth patterns, different spectrums of disease, and unusual rare genetic or congenital abnormalities.

In both adults and children, obesity is a major risk factor for a number of chronic diseases, a steady and continuous rise in prevalence over the past four decades holds serious and ominous medical and economic burdens (Kopelman, 2000). The phenotype is highly heritable. Family and twin studies have shown that between 40 and 70% of the inter-individual variation in obesity can be attributable to genetic factors (Maes et al., 1997). In recent years, large-scale GWAS have identified many loci associated with Body Mass Index (BMI), the most common measure of obesity, but these loci combined explain only 2–4% of the heritability (Speliotes et al., 2010). Thus far, four waves of GWAS studies for BMI identified 32 loci that reached genome-wide significance and unequivocally were associated with BMI in a large meta-analysis performed by the GIANT (Genetic Investigation of ANtropometric Traits) consortium (Frayling et al., 2007; Scuteri et al., 2007; Loos et al., 2008; Willer et al., 2009; Speliotes et al., 2010; Loos, 2012; Mägi et al., 2013). The firstly identified locus, FTO (fat mass and obesity associated gene), has the largest effect on obesity-susceptibility with obesity at a risk of 1.20 fold. Moreover, the frequency of the BMI-increasing allele is high in white Europeans (i.e., 40%). As a consequence, of all 32 BMI-associated loci, the FTO locus explains the largest proportion of the inter-individual variation in BMI (0.34%; Speliotes et al., 2010; Loos, 2012).

In this study, we investigated the genetic association of pediatric BMI using anthropomorphic measures extracted from medical records in a collection of already genotyped samples from two large pediatric cohort repositories (CCHMC and CHB) in order to confirm and identify additional genetic loci.

## MATERIALS AND METHODS

### STUDY SUBJECTS

Protocols for this study were approved by the Institutional Review Boards (IRBs) at the institutions where participants were recruited. Only those self-reported to have European ancestry were selected for study. The anthropometric measurements of height and weight, as well as age of measurement and gender, were extracted from the EMR. All enrolled participants with measured weight and height on the same day were included. All inconsistent or out of range values were excluded. Out of range was defined as any height or weight values higher or lower than is considered biologically possible according to Centers of Disease Control and Prevention (CDC) growth charts. Children and teens, aged 2 through 19 years old were included based on CDC growth chart requirements. In addition, three patients with the ICD-9 code for Prader Willi syndrome were excluded from final results. After removing the missing data and outliers, out of a total of 5,049 individuals, 2860 samples were included in the study. The demographic distributions of these samples are shown in **Table 1**.

**Table 1:** Demographic distribution of pediatric cohorts under study.

	# Europeans	# After removing outliers and missing values	M/F	Mean age (95% CI)	Array
CHB	741	613	387/226	13.30 (12.97–13.66)	Affymetrix-Axiom
CCHMC	829	696	338/358	10.80 (10.53–11.12)	Omni-5
	657	405	261/144	7.18 (6.73–7.63)	Omni-1
	1270	942	589/353	7.32 (7.03–7.62)	Illumina-610
	1552	204	28/176	13.70 (13.13–14.23)	Affymetrix-6
Total	5049	2860	1603/1257	9.8 (8.67–10.85)	

### GENOTYPING

High throughput single nucleotide polymorphism (SNP) genotyping was carried out previously in CCHMC and BCH using different Illumina^TM^ or Affymetrix^TM^ platforms (**Table 1**). Quality control (QC) of the data was performed before imputation. In each genotyped cohort, standard QC criteria were met and SNPs were removed if (a) >10% missing genotyping, (b) out of Hardy–Weinberg equilibrium (HWE, *P* < 0.001), or a minor allele frequency (MAF) <1%. Samples with call rate <98% were excluded. Principle component analysis (PCA) was performed to identify outliers and hidden population structure using EIGENSTRAT (Price et al., 2006). Based on examination of the scree plot, the first two PCs were retained and used as covariates during the association analysis in order to adjust for population stratification.

### PHENOTYPING

We obtained height and weight measurements from EMR in order to calculate BMI [wt (kg)/(ht(m)^2^]. When multiple measurements were available for a subject, the most recent measurement was selected and all inconsistent measures were excluded. BMI *z*-scores and percentiles were generated using the 2000 CDC growth charts (study^1^). These *z*-scores and percentiles account for the age and sex differences in BMI throughout childhood. All data for BMI-*z* scores (-3 to +3, mean = 0) were scaled to positive value (+4, 1–7, mean = 4) to be used as a quantitative trait for the GWAS study. In order to assess the burden of increased body weight on health and estimate the effect size, standard cut-offs were also used, and the tail BMI distribution was considered as a binary phenotype (≥95% as case and ≤20% as control). For the published FTO locus, Phenome wide association study (PheWas) was also performed in which presence or absence of each ICD-9 codes were considered as binary phenotype. Only ICD-9 codes with 50 or more available samples were included (143 codes) in the analysis.

### STATISTICAL ANALYSIS

Genome-wide association studies analysis was performed by cohort in PLINK (Purcell et al., 2007) using regression models and adjusting for age, sex, and the first two principal components (PC). For BMI *z*-score, the primary analysis was performed using an additive effect of the minor alleles. However, as previous BMI associations have reported better model fit with different models, recessive and dominant models were subsequently evaluated. For binary phenotypes (dichotomization of BMI using a tails approach and PheWas analyses), allelic association was assessed between cases and controls by chi-square with 1 degree of freedom (df). Allelic odds ratio (OR) and 95% confidence intervals (95% CIs) were obtained. In PheWas analyses, permutation procedure was performed using sample randomization strategy in which case and control labels are permuted randomly (×10000) in order to obtain empirical *p* values and to correct for multiple testing.

For specific target regions, imputation-based analyses were performed using the impute2-Gtool pipeline and the publicly available 1000 Genomes Project as the reference haplotype panel composed of 1092 samples (release version 2 of the 1000 Genomes Project Phase I^4^ (Howie et al., 2011). For each batch of imputation runs, the standard Markov Chain Monte Carlo (MCMC) algorithm implemented in impute-2, was used with the following threshold criteria (burnin = 10, iteration = 30, and Ne = 20000, buffer = 250 kb). A threshold of 0.90 for the posterior probability of each genotype was then applied for genotype calling and conversion using Gtool. For each imputation run, the overall genotype concordance rate was more than 95%. Additional post imputation filtering were also implemented to remove poorly imputed variants with low concordance rate according to the impute-2 standard protocol (info >0.4; Howie et al., 2011). To graphically display the results, LocusZoom was used (Pruim et al., 2010).

### META-ANALYSES

The results from primary analysis in each cohort were assembled to conduct a fixed effects weighted *Z* meta-analysis using Metal (Willer et al., 2010). This approach controls the differences in phenotype scaling across the studies and weights the signed *Z* statistics from each study by its sample size (i.e., weighted sum), from which a probability is calculated. The program also applies the genomic control correction to control type I error rates using summary statistics from each cohort. After QC filtrations in each cohort (as described above), meta-analyses were performed on SNP markers that were overlap among all five cohorts. 92670 SNP markers were in this category. At the next step in order to allow heterogeneity between cohorts, we applied a minimum weight of at least 1000 samples for analyses and identify additional effects. 583824 SNP markers were evaluated in this mode. We considered genome-wide significance thresholds of nominal *p*-value <10^-^^8^ for any new findings and report all significant results (*p* < 0.001) of previously known loci that concurred with previous publications in terms of strand direction, MAF, and supporting evidence from nearby region. In addition, to describe the presence or absence of excess variation between cohorts, we evaluated the *Q*-statistic and *I*^2^ as a measure of heterogeneity (Willer et al., 2010).

## RESULTS

The demographic distribution of the European ancestry population under study (**Table 1**) shows that the overall mean age of participants was 9.8 (95% CI = 8.67–10.85) years old with 56% being male. **Table 2** shows the estimated prevalence of overweight and obesity in these pediatric cohorts with a rate of 28% overweight (≥85th %ile) and 14% of obesity (≥95th %ile). This distribution was consistent across all cohorts (**Table 2**).

**Table 2:** Summary of BMI-for-age and prevalence of overweight and obese children from the CCHMC-BCH cohorts.

	Male	Female	Total
Number of children assessed	1603	1257	2860
Underweight (<5th %ile)	10	9	10
Normal BMI (5th–85th %ile)	62	63	62
Overweight or obese (≥85th %ile)^*^	28	28	28
Obese (≥95th %ile)	15	12	14

*Summary of children’s BMI-for-age and prevalence of overweight and obesity. *

* Terminology based on Barlow and the Expert Committee (2007).

**FIGURE 1:**
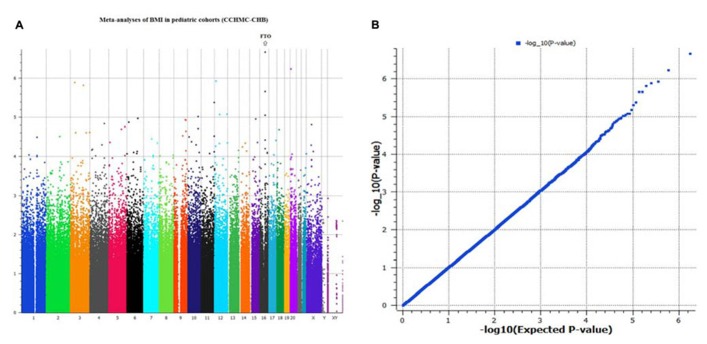
**(A,B)** Manhattan plot and *Q*–*Q* plot of SNP markers used for meta-analyses (genomic inflation, λ = 1.007).

Genome-wide analyses were conducted within each cohort. Associations between SNPs and BMI assumed an additive genetic model and summary statistics were subsequently used for meta-analysis using a weighted *z*-score method. After cleaning the data by applying our QC criteria, the ratio of the observed to expected χ^2^ test statistic (lambda) was λ = 1.007 (**Figure 1B**). The results of the meta-analyses of all studies revealed a significant signal of association at 16q12 (Manhattan plot, **Figure 1A**).

**FIGURE 2:**
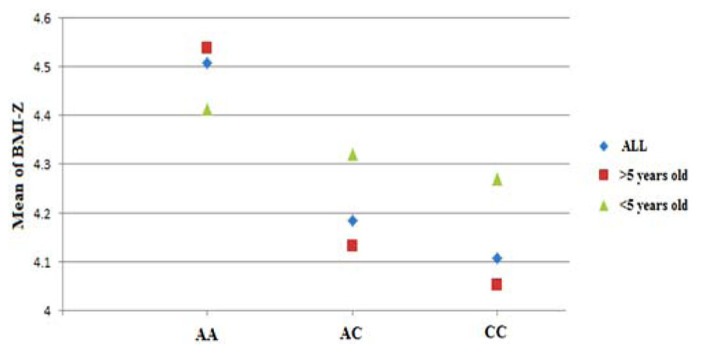
**Genotypic correlation of FTO-SNP rs8050136 with mean of BMI-*z* score.** The result further divided by age above and less than 5 years old respectively. All BMI-*z* scores (-3 to +3, mean = 0) were transformed to positive value (+4, 1–7, mean = 4).

The typed SNP rs8050136 at first intron of FTO gene produced consistent evidence of association in all cohorts with the best overall result of (*P* = 1.43 × 10^-^^7^, *z* = 5.26) and with no heterogeneity between study cohorts (*p* = 0.77; **Figure 1A**; **Table 3**). In addition, the allele frequencies and strand alignment are similar across cohorts and consistent with European ancestry. In consistent with previous publications, when mean of BMI-*z* was stratified by genotype (AA, AC, CC) for SNP (rs8050136), the additive association with risk allele was observed and it is shown in **Figure 2** (risk allele, A). There was 0.4 *z*-score-unit difference in mean of BMI-*z* score between homozygotes with risk and non-risk genotype in our pediatric cohorts (**Figure 2**). We further subdivided all cohorts into two age strata of less than 5 and above 5 years old. In meta-analyses of both strata, the minor allele (A) was associated additively with a higher BMI (**Figure 2**).

**Table 3:** Most associated SNPs with BMI-*z* in CCHMC-BCH pediatric cohorts.

Chr	Position	Gene	SNP	Minor allele	MAF	*Z* score^*^	*P*_(add_)	*P*_(rec)_	*P*_Dom_	*Cochran-q*	*I*^2^ (%)
16	53816275	FTO	rs8050136	A	0.39	5.26	1.43e-07	7.34e-08	0.0008	0.71	0
16	53813367	FTO	rs17817449	G	0.39	5.02	5.56e-07	5.10e-08	0.001	0.49	0
16	53820527	FTO	rs9939609	A	0.39	5.01	6.07e-07	8.53e-08	0.001	0.75	0
16	53800954	FTO	rs1421085	C	0.39	4.65	3.45e-06	8.21e-09	0.02	0.61	0
18	57673799	Near MC4R	rs12964056	A	0.24	-4.98	6.87e-07	0.0004	4.96e-06	0.95	0
3	42299870	CCK	rs8192472	A	0.37	-4.85	1.33e-06	0.0007	0.0002	0.89	0
4	142763570	IL15	rs2099884	T	0.15	4.29	1.27e-05	0.0006	0.0004	0.82	0
2	142855291	LRP1B	rs7583748	G	0.10	-3.81	0.0001	0.03	8.08e-005	0.19	0.41
2	644953	TMEM18	rs7561317	A	0.17	-3.17	0.001	0.09	0.02	0.74	0

* The direction of all effect (weighted z scores) are for the minor alleles. The I^2^ inconsistency metric was null to small for all of the markers (I^2^ = 0–42%).

**FIGURE 3:**
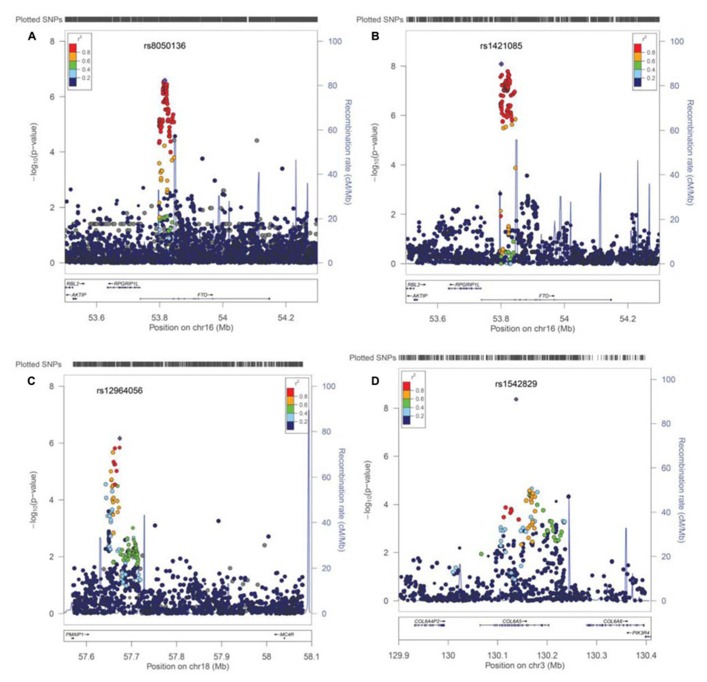
**Post imputation results on selected regions.** **(A)** Imputation results and signal at FTO locus contributing to BMI. SNPs are plotted by position in a 0.2 Mb window of chromosome 16 against association with BMI-*z* (-log10 *P*-value). The panel highlights the most significant SNP in a meta-analysis using an additive model. Estimated recombination rates (from HapMap) are plotted in cyan to reflect the local LD structure. The SNPs surrounding the most significant SNP (rs8050135), are color-coded to reflect their LD with this SNP (taken from pairwise *r*^2^ values from the HapMap CEU database, www.hapmap.org). Regional plots were generated using LocusZoom (http://csg.sph.umich.edu/locuszoom). **(B)** Regression results at the FTO locus under recessive model, best marker = rs1421085, *p*(rec) = 8.21 × 10^-^^9^. **(C)** Imputation results near the MC4R locus at chromosome 18, is shown. Best marker rs12964056, *p* = 6.87 × 10^-^^7^, *z* = -4.98. **(D)** A new effect at the COL6A5 locus in chromosome 3. Best marker rs1542829 *p* = 4.35 × 10^-^^9^, *z* = 5.889.

Next, we performed imputation-based association followed by conditional analysis to identify independent association in the FTO locus. We identified 71 SNPs at the first intronic region of FTO that were significantly associated with BMI in European ancestry, all of which were of similar magnitude (*Z* score 4.4 < *z* < 5.26, *p*-values between 10^-^^7^ < *p* < 10^-^^6^) and with high linkage disequilibrium (LD) with each other (*r*^2^ > 0.8; **Figure 3A**). The haplotype boundaries and recombination rate in this ancestry are also shown in **Figure 3**. Indeed, the SNP rs8050136 was in proxy with other well-known variants in this region including rs9939609 (*r*^2^ = 0.98), rs17817449 (*r*^2^ = 0.99), rs1421085 (*r*^2^ = 0.93) and all resided in the same haplotype and are associated with obesity in adults (4–9). Therefore, no independent effect has been identified. **Table 3** shows the summary of SNP results after regression analyses under additive and recessive models adjusted for age, gender, and PC. No significant difference was observed when the cohort site is included as another covariate (Data not shown). Of note, and only in this intron, more than 10 polymorphic indels were also associated with BMI-*z*, in particular A/AT at chr16:53822169 (MAF = 36%) and TTTC/T at chr16:53829962 (MAF = 35%; *p* = 2.41 × 10^-^^5^, *p* = 8.09 × 10^-^^5^, respectively). We noticed a subtle improvement of overall results using recessive model in our cohorts (**Figure 3B**; **Table 3**). In particular, another published SNP rs1421085 generated the best result *z* = 5.782, *p*_(__R__ec)_ = 8.21 × 10^-^^9^ (**Table 2**).

Although BMI is a continuous trait, standard cut-offs were also used to assess the burden of increased body weight on health as a binary phenotype and estimate the effect size. When the tail distribution was considered (>95% as case and <20% as control), an OR of 1.61 (95% CI = 1.31–1.97, *p* = 4.04 × 10^-^^6^) was detected for the best surrogate marker rs8050136, adjusted by age, sex, and PC. The strongest effect size was observed using a recessive model for rs1421085 [(OR = 2.79, 95% CI = 1.89–4.10), *p* = 1.83 × 10^-^^7^].

When heterogeneity was allowed between cohorts, weaker signals were also obtained in loci, such as near the MC4R region (rs12964056, *p* = 6.87 × 10^-^^7^, *z* = -4.98), cholecystokinin CCK (rs8192472, *p* = 1.33 × 10^-^^6^, *z* = -4.85), Interleukin 15 (rs2099884, *p* = 1.27 × 10^-^^5^, *z* = 4.34), low density lipoprotein receptor-related protein 1B [LRP1B (rs7583748, *p* = 0.00013, *z* = -3.81), and near transmembrane protein 18 (TMEM18; rs7561317, *p* = 0.001, *z* = -3.17)], all of which have been previously reported to be associated with obesity or BMI (**** **Table 3**; 4–10). The imputation result for the MC4R region shows multiple association markers (**Figure 3C**).

We also performed imputation in additional regions of interest. Of note, one new locus that has not been previously reported to be associated with BMI, passed the GWAS significance level in our cohorts (*p* < 1.0E-10^-^^8^). The best marker was a relatively infrequent intronic SNP, rs1542829 in the COL6A5 gene in chromosome 3, with overall MAF of 5% that produced a *p* = 4.35 × 10^-^^9^, *z* = 5.89 under an additive model adjusted for age, gender, and PC. The allele frequency of this marker was consistent among cohorts and with CEU-Hapmap data, and it was in HWE. Additional SNP markers in this region, after imputation, produced probabilities at the level of 10^-^^5^(**Figure 3D**). Of note, the majority of these SNPs had MAF of less than 10% with less cohesive haplotype boundaries (**Figure 3D**). When we considered the tail distribution (>95% as case and <20% as control) of BMI-*z* as a binary phenotype, an OR of 2.90 (95% CI = 1.93–4.34), *p* = 9.03 × 10^-^^8^ was obtained for this marker with MAF of 9% in cases vs. 3% in controls. Other unreported loci with suggestive associations to pediatric BMI (10^-^^7^ < *p* < 10^-^^5^), include KCNH5 (rs10136789, *p* = 4.62 × 10^-^^7^, *z* = 5.05), APOL5 (rs2016586, *p* = 3.26 × 10^-^6, *z* = -4.67), LRRC7 (rs10889850, *p* = 1.77 × 10^-^^6^, *z* = -4.78), and GALNT13 (rs12693973, *p* = 1.65 × 10^-^^6^, *z* = -4.80).

Finally, using ICD-9 diagnostic codes in our collections, we have also performed a Phewas study for the best identified markers in the FTO locus. In this approach, presence or absence of each diagnostic ICD-9 code was included as a binary phenotype, allelic associations were assessed between cases and controls, and the final results were corrected for multiple testing. By this approach, a negative association was detected between cases with hypertrophic cardiomyopathy or valvular structural heart disease and the FTO common risk alleles (ICD-9 codes = 424.0–424.3 and 425) in a subset of 81 cases and 2259 controls. This effect remained significant after 10000 permutations [*p*(perm) = 0.0009, OR = 0.53 (95% CI 0.37–0.77)]. Suggestive positive associations were also observed between the risk alleles and ICD-9 codes for impaired glucose tolerance test (ICD-9 = 790.2) and myopia (ICD-9 = 367.1; *p* < 0.05), however, this effect did not remain significant after permutation and correcting for multiple testing. It is noteworthy to mention that in our pediatric cohorts, the number of patients with diabetes related diagnostic codes (one of the BMI-related phenotype) was small and not sufficient for independent analysis.

## DISCUSSION

In this study, we evaluated BMI in five European ancestry pediatric cohorts with available EMR-linked genotyped data from the CCHMC-BCH eMERGE-II Network site. We successfully utilized anthropomorphic measures to calculate BMI-for-age percentile, derived BMI-*z* scores according to CDC growth charts, performed quantitative trait locus GWAS study and conducted meta analyses. The overall adjusted meta-analysis result of 2860 European samples produced the best signal in the 16q12 genomic region at the first intron of the FTO locus for a cluster of SNPs. The best typed marker rs8050136 produced [*p*_(Rec) _= 7.34 × 10^-^^8^), *z* = 5.26] and with no heterogeneity between cohorts (*p* = 0.77). When the tail distribution was considered (≥95% as case and ≤20 as control), an OR of 1.61 (95% CI = 1.313–1.965, *p* = 4.04 × 10^-^^6^) was detected. Notably, this OR estimate in our data was relatively higher than previous adult studies (OR = 1.2; Loos, 2012). In any case, considering the effect size (or OR) at the range of 1.2 and the high MAF of the FTO loci (0.40) in Europeans, 700 samples were sufficient for us to achieve an optimum power of 0.8 with a type 1 error level of 0.05. In fact, genetic variants in the first intron of FTO present as the strongest BMI-associated GWAS locus in humans. Over the past few years, the association of the FTO locus has been repeatedly replicated, not only for BMI, but also for obesity risk, body fat percentage, waist circumference, and other obesity-related traits, in particular type II diabetes (Scuteri et al., 2007; Frayling et al., 2007). Recent association with dyslipidemia, hypertension, reduced brain volume, Alzheimer’s disease, and dementia has also been reported that could be confounded with obesity and vascular complications (Pausova et al., 2009; Keller et al., 2011).

The functional mechanism of FTO however is still elusive and is currently the subject of intense interest. It is an AlkB-like, Fe(II)- and 2-oxoglutarate-dependent nucleic acid demethylase that has been shown to demethylate 3-methylthymine and 3-methyluracil in single-stranded DNA and RNA, respectively (Gerken et al., 2007). A link between FTO demethylase activity and increased fat mass was suggested by recent animal studies. Notably, homozygous mutant *fto*^-^^/^^-^ mice show postnatal growth retardation and a significant reduction in adipose tissue and lean body mass, an observation that also was supported by the deleterious mutation Ile367Phe in mouse FTO protein with an impaired activity from a separate study (Church et al., 2009; Gao et al., 2010). In both studies, the leanness of *fto*-deficient mice seems to be the result of increased energy expenditure and systemic sympathetic activation. Interpreting energy expenditure from these results, however, was challenging because of body composition differences and growth retardation. Overall, these experimental data suggest that inactivation of the FTO gene protects from obesity. On the other hand, mice globally overexpressing FTO are obese, hyperphagic, and exhibit normal energy expenditure when corrected for lean tissue mass (Church et al., 2010). Most human studies also report that obesity-predisposing FTO alleles are associated with increased food intake, but not energy expenditure (Speakman et al., 2008; Haupt et al., 2009). Although the detailed mechanisms are still not clear, this is suggested to be the result of increased expression of FTO in humans, due to cis-regulatory variation in intron 1 of this gene which is consistent with the mouse models, in which decreased levels of FTO cause a lean body habitus (Zabena et al., 2009; Berulava and Horsthemke, 2010). Moreover, a positive correlation of FTO gene expression with other adipocytokine gene expressions, including leptin, perilipin, and visfatin, has also been shown (Haupt et al., 2009).

Consistent with previous studies and because of high LD between FTO intron-1 markers in Europeans (**Figure 2**), conditional analyses didn’t produce independent effect; however, we observed a subtle improvement of FTO association using recessive models in comparison to additive models (**Figure 1**; **Table 2**). This effect was unique to the FTO locus, while use of a dominant model was best near the MC4R region (Table 3). Indeed, between-study variations in regard to the optimal inheritance model of the FTO polymorphisms have been noticed previously. In one study, eight different meta-analysis results of BMI in women with polycystic ovary syndromes were systematically reviewed and the recessive model was found to fit best in half of them and the additive model worked best in the rest (Wojciechowski et al., 2012). In our pediatrics population in which we had an enrichment of earlier age of obesity with a prevalence rate of 28%, a recessive pattern may be more relevant in comparison to the general population. Under this model, another intron-1 marker, rs1421085, produced the best result *p*_(Rec) _= 8.21 × 10^-^^9^, *z* = 5.78 (Table 3; *r*^2 ^= 0.93 with rs8050136). Indeed, the variant rs1421085 is particularly interesting. It is located within a highly conserved element and the risk allele C has been predicted to substantially reduce binding affinity for CUX1, a transcription factor implicated in the regulation of FTO (Stratigopoulos et al., 2011; Peters et al., 2013).

Limited evidence suggests that the cross-sectional FTO association with BMI varies by age. Specifically, at early ages (up to 5–7 years), the association between common variation at FTO and BMI appears to be reduced in magnitude (Hardy et al., 2010). Longitudinal twin studies also suggest that with increasing age loci such as FTO may be able to exert a greater effect on BMI which depends on the influence of shared environmental effect and the timing of adiposity rebounds (Haworth et al., 2008; Sovio et al., 2011). In our cohorts when we divide all samples into two strata of less than 5 and above 5 years old, we didn’t observe any opposing effect, however a higher magnitude was identified for strata above 5 years old (OR = 1.72) (**Figure 2**). Larger sample size with detailed longitudinal data would seem to be needed to fully elucidate this correlation.

Furthermore, in the context of rare and severe phenotypes, recently, in a large Palestinian Arab consanguineous multiplex family with nine affected, a homozygous R316Q enzyme inactivating mutation in the FTO gene, resulted in a broad spectrum of clinical manifestations including severe intrauterine growth retardation, severe microcephaly, and death from infection before the age of three (Boissel et al., 2009). Of note, six out of eight cases had structural heart defect with cardiomyopathy. This would appear consistent with our unique observation of a negative association of obesity-predisposing FTO alleles with cardiomyopathy [*p*_(corr)_ = 0.0009, OR = 0.53 (95% CI 0.37–0.77)] that could result in lower expression of FTO; although further studies with larger sample sizes are necessary to confirm or refute this finding. In fact, to our knowledge, no SNP in intron 1 of FTO has been previously associated with any trait unrelated to BMI. Recently, an independent effect at the eighth intron of the FTO locus has been reported to be associated with melanoma (GenoMEL Consortium et al., 2013). The best marker, rs16953002, was replicated using 12,313 cases and 55,667 controls of European ancestry in a study conducted by the GenoMEL consortium (combined *P* = 3.6 × 10^-^^12^, OR = 1.16). Notably, in their study, none of the BMI related SNPs in intron 1 were associated with melanoma. Similarly, in our collection, there was no effect observed at the eighth intron of the FTO gene with BMI (*p* = 0.54 for rs16953002, *r*^2^ < 0.01 with rs8050136). This suggests independent functions and genetic risks for FTO that broaden the existing paradigm and identify distinct pathogenic effects (GenoMEL Consortium et al., 2013).

In this report, we have supported association in other previously reported BMI loci, in particular loci near the MC4R region (rs12964056, *p* = 6.87 × 10^-^^7^, *z* = -4.98), cholecystokinin CCK (rs8192472, *p* = 1.33 × 10^-^^6^, *z* = -4.85), Interleukin 15 (rs2099884, *p* = 1.27 × 10^-^^5^, *z* = 4.34), low density lipoprotein receptor-related protein 1B [LRP1B (rs7583748, *p* = 0.00013, *z* = -3.81)] and near transmembrane protein 18 (TMEM18; rs7561317, *p* = 0.001, *z* = -3.17; **Table 2**). Because of their lower effect on BMI and obesity risk (OR ~1.10, in adult meta-data) and lower allele frequency, the identification of these loci requires a quadrupling of the sample size in a random population. Finding all of these loci in our pediatric collections, despite limited sample size, indicate the enrichment of the genetic signal to noise ratio given the shorter amount of time that environment has had an effect.

Additionally, we have detected a new unreported signal at chromosome 3 (Col6A5) (best SNP is rs1542829, MAF of 5% *p* = 4.35 × 10^-^^9^, *z* = 5.89). This marker produced an OR of 2.90 when the tail distributions (>95% as case and <20% as control, 386 cases, and 572 controls) was considered as binary phenotype. Considering this level of OR (2.90), even with MAF of 5%, 500 samples were sufficient for us to achieve the extraordinary power (0.99) with a type 1 error level of 0.05. This could be considered as one of the rare obesity risk loci that we expect to detect in these special cohorts. The α5-containing collagen VI (Col6A5, COL29A1), belongs to the class of collagens containing von Willebrand factor type A domains. These collagens form filaments with globular domains containing vWA motifs, which are involved in protein-ligand interactions for the organization of tissue architecture and cell adhesion. Collagen VI is a major extracellular matrix (ECM) protein with a critical role in maintaining skeletal muscle functional integrity. It has been suggested that type VI collagen is a fibrotic component that restricts adipose tissue expandability. In humans, Col6A3 gene expression in adipose tissues was found to correlate with visceral adipose tissue mass and pro-inflammatory gene expression (Pasarica et al., 2009). Mutations in different families of this gene have also been associated with myopathy and muscular dystrophy. Recently, it has been shown that COL6A5 is involved in adhesion at myotendinous and dermal–epidermal junctions (Sabatelli et al., 2012). Different polymorphisms in or near this gene have been linked to atopic dermatitis and eczema, but with contradictory reports (Söderhäll et al., 2007; Naumann et al., 2011). In our cohorts, the number of samples with Atopic dermatitis and related conditions (ICD-9 = 691) was only 49 with a trend of association for published SNP rs7629719 (*p* = 0.14); adjusting the results based on presence or absence of atopy, didn’t have any effect on overall BMI associations. A larger sample size is necessary to further elucidate this coexistent condition and to determine whether COL6A5 has any role in obesity related conditions.

Four additional novel loci with homogenous but suggestive associations (10^-^^7^ < *p* < 10^-^^5^) to childhood BMI were also identified in this study. These include KCNH5 (rs10136789, *p* = 4.62 × 10^-^^7^, *z* = 5.05), a voltage-gated potassium channel with various function in neurotransmitter regulation, hormone release, cardiac function, and cell volume; APOL5 (rs2016586, *p* = 3.26 × 10^-^^6^, *z* = -4.67), a component of high-density lipoprotein with a potential role in lipid metabolism; LRRC7 or Densin (rs10889850, *p* = 1.77 × 10^-^^6^, *z* = -4.78) a core component of post-synaptic densities and GALNT13 (rs12693973, *p* = 1.65x10^-^^6^, *z* = -4.80) a member of the UDP-*N*-acetyl-alpha-D-galactosamine and a major enzyme responsible for the synthesis of *O*-glycan. Independent cohorts are necessary to confirm these preliminary suggestive findings and their importance in childhood obesity.

Despite the limitations of using an EMR-derived data set for analysis of secondary phenotypes including errors in data extractions, discordant time of sampling, and underlying coexistent conditions of our pediatric cohorts, we demonstrate that a strong signal, larger than seen in adult populations is detectable. We have removed all inconsistent data and outliers to the best of our ability. We have also excluded infants and those less than 2 years old because of the complexity of growth chart pattern and many potential maternal effects on infants from perinatal periods. In addition we assessed the distribution of BMI-*z* in the whole population with a large sample size as a quantitative trait rather than attempting to identify limited cases and controls. From the statistical standpoint, quantitative traits usually are preferred in meta-GWAS studies because they improve power to detect a genetic effect and often have a more interpretable outcome (Bush and Moore, 2012). Furthermore, BMI is a highly heritable trait in humans and, as mentioned above, up to 70% of the inter-individual variation in obesity can be attributable to genetic factors *per se* (Maes et al., 1997); therefore, given the strong genetic confirmation described here, indeed, we managed to repurpose the genotyping data collected for the analyses of another phenotype and successfully find association between the new phenotype and genotypic data.

In summary, using the EMR-linked genotyped data, we have confirmed association of several previously known BMI loci, in particular with the FTO gene [OR of 1.61 (95% CI = 1.31–1.97)]. Our data also support the importance of variants at the FTO locus in childhood obesity and with saturation of an earlier age of onset, these data point to a closer functional variant in this locus.

## Conflict of Interest Statement

The authors declare that the research was conducted in the absence of any commercial or financial relationships that could be construed as a potential conflict of interest.

## Footnotes

1

2
